# Predicting survival in melanoma patients treated with concurrent targeted- or immunotherapy and stereotactic radiotherapy: Melanoma brain metastases prognostic score

**DOI:** 10.1186/s13014-020-01558-8

**Published:** 2020-06-01

**Authors:** Jana Schaule, Stephanie G. C. Kroeze, Oliver Blanck, Susanne Stera, Klaus H. Kahl, Falk Roeder, Stephanie E. Combs, David Kaul, An Claes, Markus M. Schymalla, Sonja Adebahr, Franziska Eckert, Fabian Lohaus, Nasrin Abbasi-Senger, Guido Henke, Marcella Szuecs, Michael Geier, Nora Sundahl, Daniel Buergy, Reinhard Dummer, Matthias Guckenberger

**Affiliations:** 1 Department of Radiation Oncology, University Hospital Zurich, Zurich, Switzerland; 2 Department of Radiation Oncology, Friedrich-Alexander-University Erlangen-Nürnberg, Erlangen, Germany; 3 University Medical Center Schleswig-Holstein, Kiel, Germany; 4 Department of Radiation Oncology, University Hospital Frankfurt, Frankfurt, Germany; 5 Department of Radiation Oncology, Universitätsklinikum Augsburg, Augsburg, Germany; 6 Department of Radiation Oncology, University Hospital Munich, Munich, Germany; 7 Department of Radiation Oncology, Technical University Munich (TUM), Munich, Germany; 8 Institute of Radiation Medicine (IRM), Helmholtz Zentrum München (HMGU), Oberschleißheim, Germany; 9 German Cancer Consortium, Partner Site Munich, Munich, Germany; 10 Department of Radiation Oncology, Charité-University Hospital Berlin, Berlin, Germany; 11 Department of Radiation Oncology, University Medical Center Utrecht, Utrecht, the Netherlands; 12 Department of Radiation Oncology, Philipps-University Marburg, Marburg, Germany; 13 Department of Radiation Oncology, Medical Center, Faculty of Medicine, University of Freiburg, Breisgau, Germany; 14 German Cancer Consortium, Partner Site Freiburg, Freiburg, Germany; 15 German Cancer Research Center (DKFZ), Heidelberg, Germany; 16 Department of Radiation Oncology, Eberhard Karls Universität Tübingen, Tübingen, Germany; 17 Department of Radiotherapy and Radiation Oncology, Faculty of Medicine and University Hospital Carl Gustav Carus, Technische Universität Dresden, Dresden, Germany; 18 German Cancer Consortium, Partner Site Dresden, Dresden, Germany; 19 Department of Radiation Oncology, University Hospital Jena, Jena, Germany; 20 Department of Radiation Oncology, Kantonsspital St. Gallen, St. Gallen, Switzerland; 21 Department of Radiation Oncology, University Hospital Rostock, Rostock, Germany; 22 Department of Radiation Oncology, Ordensklinikum Linz, Linz, Austria; 23 Department of Radiation Oncology, Ghent University Hospital, Ghent, Belgium; 24 Department of Radiation Oncology, Universitätsmedizin Mannheim, Medical Faculty Mannheim, Heidelberg University, Mannheim, Germany; 25 Department of Dermatology, University Hospital Zurich, Zurich, Switzerland

**Keywords:** molGPA, Melanoma, Stereotactic, Brain metastases, Immunotherapy, Targeted therapy

## Abstract

**Background:**

Melanoma patients frequently develop brain metastases. The most widely used score to predict survival is the molGPA based on a mixed treatment of stereotactic radiotherapy (SRT) and whole brain radiotherapy (WBRT). In addition, systemic therapy was not considered. We therefore aimed to evaluate the performance of the molGPA score in patients homogeneously treated with SRT and concurrent targeted therapy or immunotherapy (TT/IT).

**Methods:**

This retrospective analysis is based on an international multicenter database (TOaSTT) of melanoma patients treated with TT/IT and concurrent (≤30 days) SRT for brain metastases between May 2011 and May 2018. Overall survival (OS) was studied using Kaplan-Meier survival curves and log-rank testing. Uni- and multivariate analysis was performed to analyze prognostic factors for OS.

**Results:**

One hundred ten patients were analyzed. 61, 31 and 8% were treated with IT, TT and with a simultaneous combination, respectively. A median of two brain metastases were treated per patient. After a median follow-up of 8 months, median OS was 8.4 months (0–40 months). The molGPA score was not associated with OS. Instead, cumulative brain metastases volume, timing of metastases (syn- vs. metachronous) and systemic therapy with concurrent IT vs. TT influenced OS significantly. Based on these parameters, the VTS score (volume-timing-systemic therapy) was established that stratified patients into three groups with a median OS of 5.1, 18.9 and 34.5 months, respectively (*p* = 0.001 and 0.03).

**Conclusion:**

The molGPA score was not useful for this cohort of melanoma patients undergoing local therapy for brain metastases taking into account systemic TT/IT. For these patients, we propose a prognostic VTS score, which needs to be validated prospectively.

## Background

About 40–60% of melanoma patients will develop brain metastases during the course of their disease, which contributes to a worse prognosis and quality of life [1]. Median overall survival (OS) from presentation of brain metastases to death used to be only 3 months [2]. Recent developments in the fields of targeted- and immunotherapy (TT/IT), as well as the now widespread use of stereotactic radiosurgery (single fraction, SRS) and stereotactic radiotherapy (fractionated stereotactic radiotherapy, SRT) have significantly improved survival [3]. In the following, the term SRT will be used for both, single- and multi-fraction stereotactic radiotherapy. Despite response rates of melanoma brain metastases of up to 40–60% to IT and TT, presence of brain metastases remains a limiting factor for survival [4–8], indicating that IT/TT as monotherapy is often not sufficient. SRT is effective in controlling brain metastases, with the advantage of preserving cognitive function compared to whole brain radiotherapy (WBRT) [9–11]. For a limited number of up to four brain metastases, SRT without WBRT is now the preferred treatment option [12]. However, evidence is emerging that not number, but the volume of brain metastases determines whether patients should receive SRT [10]. In the rapidly changing setting of treatment opportunities for melanoma patients it remains unclear, which patients benefit most from local treatment and there is a need for a reliable prognostic score to tailor treatment.

Several scores have been developed aiming to predict survival in patients with brain metastases [13–15]. However, caution is required as these scores have not been established or validated in patients treated with combined SRT and TT/IT. In 2010, Sperduto et al. presented a refined version of the Graded Prognostic Assessment (GPA) focusing on melanoma patients (ds-GPA) [16]. Karnofsky performance status (KPS) and number of brain metastases were identified as prognostic factors for OS. Recently, the group updated the score in the light of new molecular markers, named molGPA score [17]. In addition to the above-mentioned factors, age, BRAF-mutational status and presence or absence of extracranial metastases were added as prognostic markers. The score is four-tired as its predecessor and reflects the success of the advances in treatment by markedly prolonged median OS as compared to the ds-GPA [17]. However, radiation treatment consisted of a multitude of different treatment regimens including surgery + SRT/WBRT, WBRT +/− SRT, or SRT alone. Furthermore, the score did not take into account whether and what systemic treatment these patients received as background to local treatment. In the only study to date that validated the molGPA score, 70% of patients received WBRT and concurrent systemic therapy was likewise not recorded [18]. Other available scores have been shown to underestimate OS as these were all developed before widespread implementation of TT/IT [19]. Two studies have aimed to validate prognostic scores in TT/IT-treated patient cohorts that received concomitant SRT to date, albeit omitting the most widely used molGPA score [20, 21]. Hence, this study aims to validate the molGPA score in a cohort of patients homogeneously treated with SRT and concurrent TT/IT to depict patterns of care of modern melanoma treatment.

## Methods

A retrospective international multicenter registry study (TOaSTT) was established to collect data on patients receiving SRT (delivery of up to 10 high-dose stereotactic radiation fractions) with concurrent targeted- or immunotherapy. Ethics approval was obtained by the lead ethics committee in Zurich (BASEC-Nr. 2016–01807) and from all participating centers at their local ethics committees. For sub- analysis of melanoma patients with brain metastases, patients meeting the following criteria were included: ≥18 years of age, histopathologically confirmed diagnosis of melanoma with synchronous or metachronous (> 6 months after first diagnosis) brain metastases, patients receiving SRT of any number of cerebral metastasis and concurrent treatment with any type of the following targeted drugs: antibodies and tyrosine kinase inhibitors (targeted therapies, TT) and immune-checkpoint inhibitors (immunotherapy, IT). “Concurrent” was defined as application of the respective drug within 30 days prior or after SRT. SRT was defined as delivery of single or fractionated large dose irradiation fractions ranging from 1 × 18 Gy to 1 × 21 Gy for SRS or from 5 × 5 Gy to 6 × 5 Gy for SRT; Patients treated with linac-based SRT, robotic-SRT (Cyberknife) as well as Gammaknife were eligible. Patients receiving concurrent whole brain radiotherapy (WBRT) were excluded as well as melanoma patients in the database that only received stereotactic radiotherapy extracranially. Patients were included irrespective of prior treatment regimens.

Overall survival (OS) was defined as time of start of SRT to time of death, living patients were censored at the date of last follow-up. In the case of multiple cerebral stereotactic irradiations, OS was calculated from start of last stereotactic treatment.

The score by Sperduto et al. was applied and groups were formed as described in the original publication awarding points for age, KPS, extracranial metastases, BRAF-status and number of brain metastases [17]. After performing uni- and multivariate analyses for patient-, tumor- and treatment factors associated with OS, we established a new prognostic score using factors of statistical significance.

Descriptive statistical analysis was performed with SPSS v25.0 statistic software package (IBM Corp., Armonk, NY, USA). The Kaplan-Meier method followed by log-rank analysis for comparison of subgroups was used to evaluate OS. Univariate and multivariate Cox regression analysis (Enter method) was performed to identify independent variables for OS. Chi-square test was used to compare differences between two independent groups. A *p*-value of less than 0.05 was deemed statistically significant.

## Results

### Patient and treatment characteristics

Patient and treatment characteristics are shown in Table 1.

**Table 1:** Patient and treatment characteristics

*n* = 110 patients	Median / n (% / range)
**Age**	59.4 (25–82)
**Sex**	
Male	64 (58.2)
Female	44 (44.0)
**ECOG**	
0	77 (70.0)
1	26 (23.6)
2	6 (5.5)
3	1 (0.9)
**Number of brain metastases**	median: 2 (1–30)
1	47 (42.7)
2–4	53 (48.1)
> 4	10 (9.0)
**BRAF status**	
positive	57 (48.2)
negative/unknown	46 (41.8) / 7 (6.3)
**extracranial metastases**	
yes	83 (75.5)
no	27 (24.5)
**volume of brain metastases**	
≥ 1.5 cc	55 (50.0) 1.5–24.54 cc
< 1.5 cc	55 (50.0) 0.05–1.4 cc
**RT regimen**	
SRS	95 (86.4)
SRT	15 (13.6)
**Concomitant systemic therapy**	
IT	67 (60.9)
TT	34 (30.9)
combination	9 (8.2)

Abbreviations: *SRS* Stereotactic radiosurgery, *SRT* Stereotactic radiotherapy, *IT* Immunotherapy, *TT* Targeted therapy

One hundred ten patients were eligible for sub-analysis form the TOaSTT database. Median age at time of radiation was 59.4 years (25–82 years). The large majority of patients had a good performance status of ECOG 0–1 (93.6%). The median number of SRT-treated brain metastases was 2 (1–30). Forty-seven patients had a single metastasis, 53 had 2–4 metastases and 10 patients had > 4 brain metastases. The large majority of patients had extracranial metastases at time of cerebral SRT (83 patients; 75.5%), while only 27 patients (24.5%) suffered from exclusively cerebral disease. The median cumulative brain metastases volume was 1.5 cc ranging from 0.05–24.5 cc.

Fifty-seven (48.2%) patients harbored BRAF-mutations, 53 (51.8%) did not or it was unknown.

No Patients were treatment-naïve: 92% had received surgery (mostly for the primary tumor), 19% had received conventional immunotherapy (IL-2 or interferone), 23.6% had received conventionally fractionated radiotherapy, 2.7% had been treated with conventional chemotherapy, 38% had received previous SRT to the brain. In those cases, the last SRT was included in this analysis.

The majority of patients received concurrent IT alone (60.9%). Of those, 25.5% of patients received pembrolizumab, 16.3% ipilimumab, 12.7% nivolumab, the remaining 8% were treated with combinations of IT. Of all patients, 30.9% were treated with targeted therapy alone: Dabrafenib/trametinib was administered in 9% of cases, 8% received vemurafenib. The remaining nine patients received BRAF and/or MEK inhibitors (dabrafenib, binimetinib, trametinib, vemurafenib/cobimetinib, vermurafenib/osimertinib, encorafenib/binimetinib). Nine patients were treated with a combination of IT and TT. TT/IT was paused during SRT in 47% of patients. It was paused a median of 5 days (1–28 days) before SRT and restarted a median of 7 days (1–25 days) after SRS/SRT. Total median pause length was 14 days (1–29 days). In 49% of patients, TT/IT was not paused during SRT, data was missing for four patients. Patients treated with SRS received a median of 20 Gy in one fraction (*n* = 95; range 18-21Gy). Patients treated with SRT received 25-30Gy total dose (*n* = 15; regimens varied between 5x5Gy, 6x5Gy or 5x6Gy). Patients were treated with c-arm-based SRT with linear accelerators, robotic-based SRT (CyberKnife, Accuray, Sunnyvale, USA) as well as Gamma Knife (Elekta, Stockholm, Sweden). Median follow-up was 8 months (0–39 months) and median OS was 8.4 months (0–40 months).

### Survival analysis by molGPA score factors

The prognostic factors of the molGPA score were analyzed. These are age, performance status, presence of extracranial disease, BRAF-status, and the number of brain metastases: OS between patients older or younger than 70 years did not differ (*p* = 0.98). Performance status did influence survival on univariate, but not on multivariate analysis (Table 2).

**Table 2:** Uni- and multivariate analysis (Cox regression, Enter method)

	**n**	***Univariate***	***P*-value**	***Multivariate*** ***Enter***	***P*-value**
		**HR (death) (95% CI)**		**HR (death) (95% CI)**	
**Age**^**a**^					
< 70	82	0.99 (0.56–1.76)	0.98	0.76 (0.38–1.5)	0.43
≥ 70	27				
**ECOG**^**a**^					
0	77	0.46 (0.27–0.77)	**0.002**	0.68 (0.39–1.19)	0.18
> 0	33				
**BRAF**^**a**^					
Mutated	56	0.56 (0.34–0.93)	**0.02**	0.58 (0.32–1.05)	0.07
Wild type/unknown	53				
**Number of brain metastases**^**a**^					
1	47	1.25 (0.85–1.82)	0.29	1.5 (0.425–4.98)	0.30
2–4	52				
> 4	10				
**Extracranial metastases**^**a**^					
present	82	0.42 (0.56–1.76)	**0.02**	1.35 (0.37–4.98)	0.65
absent	27				
**Brain metastases volume**					
< 1.5 cc	54	0.56 (0.34–0.94)	**0.03**	0.54 (0.29–0.96)	**0.04**
≥ 1.5 cc	55				
**Timing**					
Synchronous	25	1.84 (1.05–3.24)	**0.03**	2.43 (1.24–4.75)	**0.01**
metachronous	84				
**Systemic therapy**					
IT	67	1.99 (0.37–8.48)	**0.001**	3.0 (0.98–9.26)	**0.005**
IT	34				
combination	8				
**Other metastases controlled**					
No	38	0.25 (0.01–1.97)	**0.001**	0.15 (0.01–12.18)	0.78
yes	34				
mixed	17				
no other metastases	19				

Abbreviations: *HR* Hazard ratio, *CI* Confidence interval; ^a^ = factors in the original molGPA score

Patients with extracranial disease had a significantly worse OS compared to patients without extracranial disease in univariate analysis (*p* = 0.02) but not on multivariate analysis. Patients harboring BRAF-mutations had a worse OS compared to patients with an unknown or no BRAF-mutation (*p* = 0.02). Median OS in the patient group with BRAF-mutations was 5.4 months whereas median OS for BRAF-wild type patients (or with an unknown BRAF-status) was 10.7 months (*p* = 0.001). Importantly, the ECOG performance status did not differ between the BRAF positive and negative/unknown groups (*p* = 0.5). The number of SRT-treated brain metastases did not influence OS (*p* = 0.29) (1 vs. 2–4 metastases *p* = 0.12, 1 vs. > 4 metastases *p* = 0.07; 2–4 vs > 4 metastases *p* = 0.68).

Univariate analysis was additionally performed for cumulative brain metastases volume, timing of metastases (syn- vs. metachronous), type of systemic therapy (IT vs. no IT) and whether other metastases were controlled at time of SRT. In contrast to the number of brain metastases, OS was significantly associated with the cumulative brain metastases volume: patients with a cumulative brain metastases volume of < 1.5 cc had a median OS of 15.5 months, while the median OS for ≥1.5 cc brain metastases volume was 6.1 months (*p* = 0.02). On multivariate analysis, only cumulative brain metastases volume, timing of metastases and type of systemic treatment remained statistically significant (Table 2).

### Application of the molGPA score and the VTS score

**Fig. 1:**
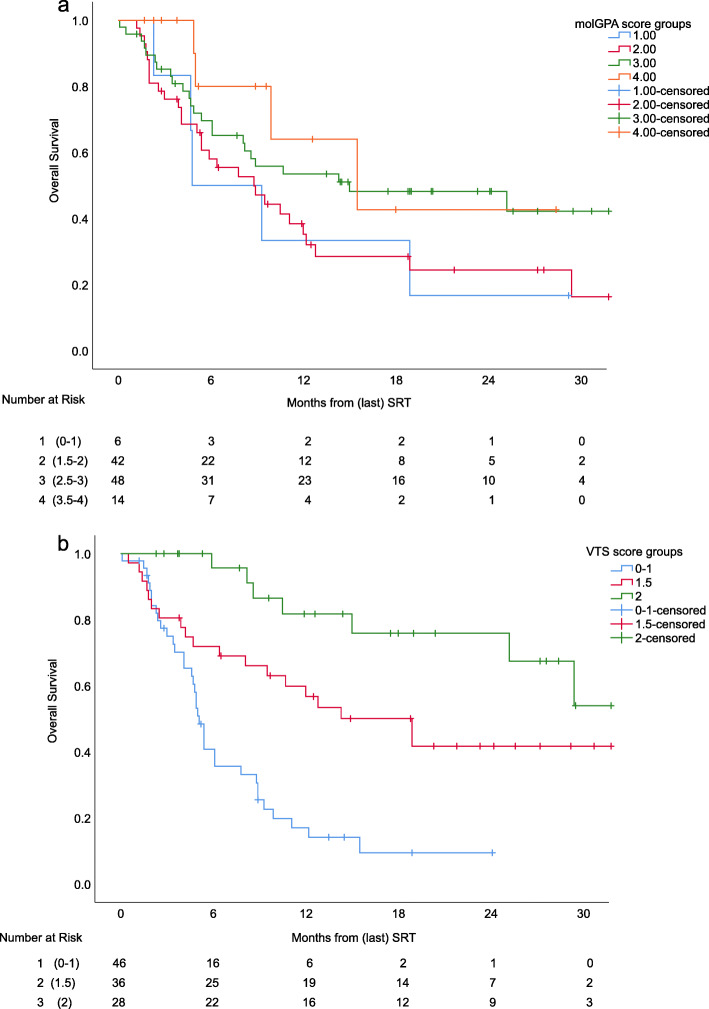
Kaplan-Meier curves comparing (**a**) the molGPA score with (**b**) the VTS score including cumulative brain metastases volume, timing of metastases, cumulative brain metastases volume and concomitant systemic treatment

The molGPA score was applied to the cohort as described by the authors to estimate survival. Median OS was 4.8, 8.8, 15.0 and 15.5 months for the four groups with 0–1 points, 1.5–2 points, 2.5–3 point and 3.5–4 points, respectively. The score did not differentiate between the four groups well (log-rank across strata *p* = 0.1, Fig. 1a).

Based on the findings of the analysis, a different score, named the VTS score was established, where cumulative brain metastasis volume, timing of metastases and type of systemic treatment were included as described above (Tables 3 and 4).

**Table 3:** Survival of patients grouped by the VTS score

**VTS score**	***n*** **= 110**	**median OS (months)**	**logrank pairwise**
0–1	46	5.1	**vs. 1.5: 0.001** **vs. 2: 0.0001**
1.5	36	18.9	**vs. 2: 0.03**
2	28	34.5	

Abbreviations: *VTS* Volume-timing-systemic therapy, *OS* Overall survival

**Table 4:** Awarded points for VTS score

	**0 points**	**0.5 points**	**1 point**
Cumulative brain metastases volume	> 1.5 cc	< 1.5 cc	
Timing	synchronous	metachronous	
Systemic treatment	targeted therapy		immunotherapy

The groups were formed as described in a three-tiered manner. The VTS score was significantly associated with OS (*p* < 0.0001, Fig. 1b) with a median OS in the three groups of 5.1, 18.9 and 34.5 months, respectively.

**Fig. 2:**
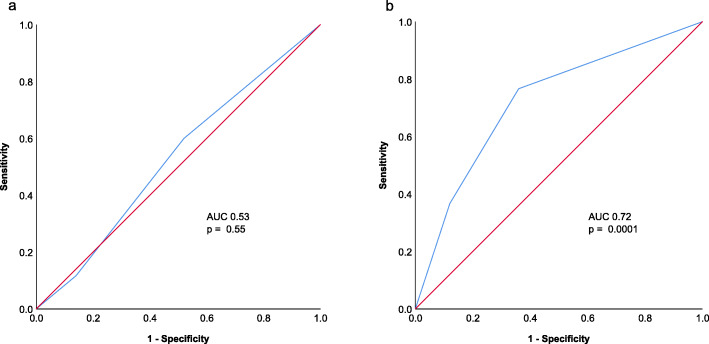
Receiver operating curves (ROC, red line = diagonal reference line) for (**a**) the molGPA score and (**b**) the VTS score for the binary endpoint of 6-months-OS with 60 patients reaching this endpoint. The AUC was improved with the VTS score

When analyzing the sensitivity and specificity of the two scores to predict survival of patients at 6 months, the molGPA score did not show the capacity to predict survival, whereas the VTS score improved the prediction significantly. Hence, in a cohort of patients treated homogenously with concurrent IT/TT and SRT, other factors than those informing the molGPA score may be of improved prognostic power (Fig. 2).

## Discussion

To our knowledge, this is the first study attempting to validate the molGPA score in a cohort of melanoma patients with brain metastases, which was homogeneously treated with concurrent SRT and targeted- or immunotherapy. As these treatment combinations are rapidly implemented in routine practice, it is crucial to validate this score or develop more appropriate ones for this patient cohort. Using the measures proposed by Sperduto and colleagues [17], we were unable to validate the molGPA score. However, when using the factors cumulative brain metastasis volume, timing of brain metastases development and type of systemic treatment, survival was stratified in a statistically significant manner. On analysis using the receiver-operating-curve for the binary endpoint of 6-months-OS, the VTS score outperformed the molGPA with an AUC of 0.74 vs. 0.53.

### Concurrent systemic treatment and SRT

Retrospective studies have investigated concurrent treatment of TT/IT and SRT in melanoma patients to date. Median OS was 7.5 months for concomitant CTLA-4-inhibitors, 17.8 months for concomitant BRAF-inhibitors and 20.4 months for concomitant anti-PD-1 treatment in a retrospective study of 108 melanoma patients receiving SRT for brain metastases (notably, 40% of patients received an additional WBRT) [22]. Gaudy-Marqueste et al. treated all brain metastases upfronct with SRT followed by IT/TT. The cohort of 179 patients showed a median OS of 10.9 months [3]. Recently, 80 patients treated with concurrent ipilimumab or nivolumab and SRT to melanoma brain metastases were evaluated retrospectively for progression-free survival, OS and toxicity profile [23]. The OS times in the present study are in line with these studies.

In our cohort, treatment with immunotherapy was a factor positively associated with OS compared to concomitant use of targeted therapy. Only two prospective phase I studies are available at the moment evaluating the safety of concurrent SRT and nivolumab and ipilimumab, respectively [24, 25]. It has been hypothesized that SRT has the propensity to synergize with IT through five factors: 1) inducing immunogenic cell death by generating antigens 2) promoting antigen presentation on MHC I molecules enhancing the tumor cell killing through CD8 cytotoxic T-cells, 3) increasing blood-brain-barrier-permeability, 4) inducing chemokines that help overcome T-cell exclusion from the metastases and 5) through the rare abscopal effect [26–28]. A retrospective analysis of 395 patients with advanced melanoma in Switzerland likewise showed an improved median OS for patients receiving IT compared to TT (16.7 months vs. 11.2 months) [29]. However, only few prospectively collected datasets have retrospectively investigated this potential synergy to date [24, 30, 31].

It currently remains unclear whether BRAF-mutated patients should receive TT or IT as first line therapy. It has been suggested that the type of BRAF-mutation (V600E vs. V600K) influences treatment response: The V600E mutation is more prone to responding to TT, while V600K-mutated tumors typically employ a higher tumor mutational burden and respond better to IT [32]. In this study, DNA-expression profiles to detect these differences were not available but should be investigated in the future to tailor treatment. There are multiple trials investigating the IT-TT combination treatment for BRAF-mutated melanoma patients showing overall response rates of up to 100%, albeit with 22% of patients discontinuing treatment due to toxicity (COMBI-I trial).

### BRAF-mutation associated with worse OS

It was surprising that the BRAF-mutated patients in our cohort had a significantly worse OS than BRAF-wildtype patients. This might be explained by the effects of IT: patients might have received IT at an earlier time point which then achieved improved OS [3]. Additionally, it has been acknowledged, that the timing of IT/TT and SRT is crucial [12, 33]. Recently, the immunomodulatory effects of SRT has been discussed in the literature leading to synergistic effects of the combined treatment as discussed above that might also be reflected in our finding [28, 34]. In the original molGPA study, BRAF-status was divided into three groups: positive, negative and unknown; whereby the unknown status (29% of patients) yielded the highest hazard ratio (1.94 vs 1.3 for negative and 1.0 for positive); nevertheless, negative and unknown were awarded 0 points in the score, raising doubts about the strength of prognostic value of this factor. In contrast, our data was very complete regarding BRAF-mutational status: the BRAF-status was unknown for only 7 patients.

### The cumulative brain metastases volume factor

The first prognostic score index for radiosurgery (SIR) for brain metastases treated with SRT (including 10 melanoma patients; 15.3%) already included a volume factor stratifying by the largest irradiated lesion in three groups (< 5 cc, 5-13 cc and > 13 cc) [35]. SIR also considered the number of brain metastases. By using the cumulative brain metastases volume, parts of both factors are combined. A more recent evaluation of multiple prognostic scores (omitting the most recent molGPA score) by Badakhshi et al. had shown the cumulative brain metastases volume to be of importance. However, while yielding the highest level of statistical significance on univariate analysis, cumulative brain metastases volume did not remain a statistically significant prognostic factor on multivariate analysis. The authors likewise stratified patients by smaller or larger than median cumulative brain metastases volume, which was similar to the median cumulative brain metastases volume in this study (2.47 cc) [20]. In their cohort of 80 patients, only 8.8% of patients received concurrent TT/IT. An evaluation of prognostic scores in 66 melanoma patients treated with SRT-only for brain metastases further supports the hypothesis that cumulative brain metastases volume contains more prognostic power compared to the mere number: on multivariate analysis, age > 60 years, performance status ≤80%, and notably, cumulative brain metastases volume > 2 cc was associated with worse OS, while number of brain metastases was not [36].

Of note, the authors of the molGPA score had investigated volume of brain metastases, although merely for SRS-only treated patients in their cohort (56%). It is not reported, whether this was the cumulative volume. Additionally, the brain metastases volume factor was applied to the entire cohort, albeit 25% of patients received WBRT, which could explain why this factor was not statistically significant for survival of the entire cohort [17].

### Limitations

Limitations of this study include its retrospective character, the small number of patients compared to the original molGPA publication, and the utilization of the ECOG score rather than the smaller scaled KPS. In the future, the newly developed VTS score needs to be externally validated. Notably, only 3.6% of patients received concurrent ipilimumab/nivolumab as systemic treatment with SRT, which is the standard of care treatment as of today [5, 6], which may be due to the recruitment time between 2011 and 2018. Combinations of BRAFi and MEKi were applied in 16.3% of cases, a standard TT-treatment for BRAF-mutated cutaneous melanoma according to ESMO guidelines [37]. FFig.

## Conclusion

In conclusion, this study shows for the first time, that the molGPA score has to be used with caution for the rapidly growing cohort of melanoma patients treated with SRT concurrent with immuno- or targeted therapies for brain metastases. Factors such as cumulative brain metastasis volume, timing of metastases and type of systemic treatment should be taken into account in this cohort. In the future, timing of TT/IT and SRT should be investigated further and the VTS score should be validated to enrich the clinical decision-making tool enabling physicians to make the best decisions regarding treatment of brain metastases for their melanoma patients.

## Data Availability

The dataset(s) supporting the conclusions of this article is (are) included within the article (and its additional file(s)).

## Abbreviations

AUC: Area under curve
ECOG: Eastern cooperative oncology group
molGPA: molecular graded prognostic assessment
IT: Immunotherapy
KPS: Karnofsky performance score
OS: Overall survival
SRT: Stereotactic radiotherapy
SRS: Stereotactic radiosurgery
TT: Targeted therapy
VTS: Volume – timing – systemic therapy
WBRT: Whole brain radiotherapy
