# Correction to: Predicting survival in melanoma patients treated with concurrent targeted- or immunotherapy and stereotactic radiotherapy: Melanoma brain metastases prognostic score

**DOI:** 10.1186/s13014-020-01708-y

**Published:** 2020-12-14

**Authors:** Jana Schaule, Stephanie G. C. Kroeze, Oliver Blanck, Susanne Stera, Klaus H. Kahl, Falk Roeder, Stephanie E. Combs, David Kaul, An Claes, Markus M. Schymalla, Sonja Adebahr, Franziska Eckert, Fabian Lohaus, Nasrin Abbasi-Senger, Guido Henke, Marcella Szuecs, Michael Geier, Nora Sundahl, Daniel Buergy, Reinhard Dummer, Matthias Guckenberger

**Affiliations:** 1 Department of Radiation Oncology, University Hospital Zurich, Zurich, Switzerland; 2 Department of Radiation Oncology, Friedrich-Alexander-University Erlangen-Nürnberg, Erlangen, Germany; 3 Department of Radiation Oncology, University Medical Center Schleswig-Holstein, Kiel, Germany; 4 Department of Radiation Oncology, University Hospital Frankfurt, Frankfurt, Germany; 5 Department of Radiation Oncology, Universitätsklinikum Augsburg, Augsburg, Germany; 6 Department of Radiation Oncology, University Hospital Munich, Munich, Germany; 7 Department of Radiation Oncology, Technical University Munich (TUM), Munich, Germany; 8 Institute of Radiation Medicine (IRM), Helmholtz Zentrum München (HMGU), Oberschleißheim, Germany; 9 German Cancer Consortium, Partner Site Munich, Munich, Germany; 10 Department of Radiation Oncology, Charité-University Hospital Berlin, Berlin, Germany; 11 Department of Radiation Oncology, University Medical Center Utrecht, Utrecht, The Netherlands; 12 Department of Radiation Oncology, Philipps-University Marburg, Marburg, Germany; 13 Department of Radiation Oncology, Medical Center, Faculty of Medicine, University of Freiburg, Freiburg im Breisgau, Germany; 14 German Cancer Consortium, Partner Site Freiburg, Freiburg, Germany; 15 German Cancer Research Center (DKFZ), Heidelberg, Germany; 16 Department of Radiation Oncology, Eberhard Karls Universität Tübingen, Tübingen, Germany; 17 Department of Radiotherapy and Radiation Oncology, Faculty of Medicine and University Hospital Carl Gustav Carus, Technische Universität Dresden, Dresden, Germany; 18 German Cancer Consortium, Partner Site Dresden, Dresden, Germany; 19 Department of Radiation Oncology, University Hospital Jena, Jena, Germany; 20 Department of Radiation Oncology, Kantonsspital St. Gallen, St. Gallen, Switzerland; 21 Department of Radiation Oncology, University Hospital Rostock, Rostock, Germany; 22 Department of Radiation Oncology, Ordensklinikum Linz, Linz, Austria; 23 Department of Radiation Oncology, Ghent University Hospital, Ghent, Belgium; 24 Department of Radiation Oncology, Universitätsmedizin Mannheim, Medical Faculty Mannheim, Heidelberg University, Mannheim, Germany; 25 Department of Dermatology, University Hospital Zurich, University Zurich, Zurich, Switzerland

## Correction to: Radiation Oncology(2020) 15:135 https://doi.org/10.1186/s13014-020-01558-8

Following publication of the original article [1], we have been notified that there is a mistake in the affiliations of authors initially mentioned. Correct affiliations of the authors are provided in this article.
